# Sustained clinical benefit of idursulfase beta in mucopolysaccharidosis II: two-year experience from a phase 3 extension study including patients switched from idursulfase

**DOI:** 10.1186/s13023-026-04492-2

**Published:** 2026-07-09

**Authors:** Minji Im, Ari Song, Hyojung Park, Jiyeon Kim, Chiwoo Kim, Eu-Seon Noh, Ga young Bae, Juyoung Sung, JiHoon Hwang, Yoon Ji Ahn, Haein Kim, Youngsin Oh, Bogyeong Kim, Sung Yoon Cho

**Affiliations:** 1 Department of Pediatrics, Samsung Medical Center, Sungkyunkwan University School of Medicine, 81 Irwon-ro, Gangnam-gu, Seoul, 06351 Republic of Korea; 2 Medical Division, GC Biopharma Corp, Yongin, Republic of Korea; 3 Present Address: Department of Pediatrics, Samsung Kiul Growth Pediatric Clinic, Namyangju-si, Gyeonggi-do Republic of Korea; 4 Present Address: Department of Pediatrics, Seongnam Citizens Medical Center, Seongnam-si, Gyeonggi-do Republic of Korea; 5 Present Address: Department of Pediatrics, Sungae Hospital, Seoul, Republic of Korea; 6 Present Address: Department of Pediatrics, VIC 365 Pediatrics Clinic, Incheon, Republic of Korea; 7 Present Address: Department of Pediatrics, Kangdong Sacred Heart Hospital, Seoul, Republic of Korea; 8 Present Address: Department of Pediatrics, LaLa Pediacric Clinic, Seongnam-si, Gyeonggi-do Republic of Korea; 9 Present Address: Department of Pediatrics, Best Children’s Hospital, Hwaseong-si, Gyeonggi-do Republic of Korea; 10 Present Address: Department of Pediatrics, Leebora Pediatrics Clinic, Uijeongbu-si, Gyeonggi-do Republic of Korea

**Keywords:** Idursulfase beta, Mucopolysaccharidosis II, Hunter syndrome, Hunterase, Enzyme replacement therapy

## Abstract

**Background:**

Long-term safety and efficacy data on enzyme replacement therapy, particularly switching between recombinant iduronate-2-sulfatase formulations, remain limited in mucopolysaccharidosis II (MPS II). This open-label extension study included male patients with MPS II who completed a 52-week, phase 3 trial and either continued idursulfase beta (maintenance group, *n* = 22) or switched from idursulfase (switch group, *n* = 6). Participants received weekly intravenous idursulfase beta (0.5 mg/kg) for an additional 52 weeks. Safety, immunogenicity, six-minute walk test (6-MWT), and urinary glycosaminoglycan (GAG) levels were evaluated longitudinally for two years. Changes over time were analyzed using descriptive statistics and mixed models for repeated measures.

**Results:**

Over the two-year treatment period, no new safety signals were identified in both groups. In the maintenance group, the incidence of adverse drug reactions decreased in the second year compared with the first year (from 54.5% to 13.6%); anti-drug antibody positivity peaked within the first six months and subsequently declined, with a low prevalence of neutralizing antibodies (4.5–22.7%). At Month 24, least-square mean changes from baseline demonstrated a 23.1% increase in 6-MWT distance and a 73.1% reduction in urinary total GAG levels in the maintenance group. Patients switching from idursulfase showed comparable safety profiles and sustained functional and biochemical improvements.

**Conclusions:**

These results demonstrate that idursulfase beta is safe and efficacious in the long-term management of MPS II. Additionally, switching from idursulfase to idursulfase beta preserved therapeutic efficacy without additional safety or immunogenicity concerns, supporting idursulfase beta as a viable option following prior idursulfase therapy.

**Registration:**

The study was registered at ClinicalTrials.gov (identifier, NCT07344376 date of registration, 7 January 2026).

**Supplementary Information:**

The online version contains supplementary material available at https://doi.org/10.1186/s13023-026-04492-2.

## Background

Mucopolysaccharidosis II (MPS II, Hunter syndrome) is an X-linked recessive disorder caused by pathogenic variants in the *IDS* gene, resulting in a deficiency of iduronate-2-sulfatase (I2S) [1, 2]. This enzymatic deficiency leads to progressive accumulation of glycosaminoglycans (GAG), ultimately causing multisystemic dysfunction [3–6]. Manifestations of MPS II include growth restriction, skeletal abnormalities, coarse facial features, joint stiffness, hepatosplenomegaly, recurrent infections, neurological complications, upper airway obstruction, and valvular heart disease. The natural course of the disease phenotype can differ, ranging from a severe early-onset form, with progressive cognitive impairment and preterm mortality in the first or second decade of life, to a milder, attenuated variant with minimal neurological involvement and survival into adulthood [1, 2]. The incidence of MPS II is estimated at < 1 per 100,000 live births, with male sex being the primary risk factor [2, 7–12]. According to epidemiological data, MPS II is the most common type of mucopolysaccharidosis diagnosed in East Asian countries, including Korea, Japan, China, and Taiwan [13]. The discovery of enzyme replacement therapy (ERT) constituted a breakthrough in the management of MPS II, previously treated solely in a palliative setting. The two recombinant enzymes, idursulfase and idursulfase beta, have been developed to replace deficient I2S activity [11]. Idursulfase (Elaprase^®^, Shire Human Genetic Therapies, Lexington, MA, United States) was approved for the management of MPS II in the United States in July 2006. The efficacy and safety of idursulfase were verified in a series of clinical trials in which the drug, infused intravenously (IV) at 0.5 mg/kg weekly, led to a significant decrease in urinary GAG excretion, reduction of hepatosplenomegaly, and substantial improvement in 6-minute walk test (6-MWT) distance [14–16]. Idursulfase beta (Hunterase^®^, GC Biopharma Corp., Gyeonggi-do, Korea) was granted approval for the management of MPS II in January 2012. Idursulfase beta shares an amino acid structure with idursulfase but has a different glycosylation pattern because it is produced in CHO-DG44 cell lines in serum-free media [17, 18]. The drug was shown to effectively reduce urinary GAG excretion and to prevent central nervous system (CNS) damage in a mouse model of MPS II [19]. The safety of idursulfase beta (0.5 mg/kg weekly, IV) and its efficacy in terms of urinary GAG excretion decrease, organomegaly reduction, and 6-MWT distance improvement were further successfully verified in phase 1/2 and 3 trials [20, 21]. In addition, another phase 1/2 trial showed that intracerebroventricular treatment with idursulfase beta (up to 30 mg monthly) was tolerated well and effectively improved neurological symptoms in children with neuronopathic MPS II [22, 23].

Data on the long-term safety and sustained efficacy of idursulfase beta beyond one year remain limited, and evidence regarding clinical outcomes after switching from idursulfase to idursulfase beta is particularly scarce. This open-label extension study is the first to report two-year results, including a switch cohort. We aimed to evaluate the long-term safety and efficacy of idursulfase beta over two years, including clinical outcomes of patients switched from idursulfase to idursulfase beta for the treatment of MPS II.

## Methods

### Study design and participant

This open-label, single-cohort study was an extension of the phase 3 trial of idursulfase beta [20]. The phase 3 trial, conducted in male participants with MPS II, who were five years and older and previously untreated for the condition, consisted of two consecutive parts: part 1, a double-blind, randomized study assessing the idursulfase and idursulfase beta groups for 52 weeks, and part 2, an open-label, single-cohort study assessing idursulfase beta group for 52 weeks. Regardless of the treatment group assigned in the phase 3 trial, participants who completed either part of the phase 3 trial were asked to consent to remain in the study to receive idursulfase beta for another year. Patients with MPS II who had been treated with idursulfase beta outside the phase 3 trial for at least six months were also eligible for the extension study if the person were a male and aged five years or older. Fertile participants were required to use effective appropriate contraceptive methods during the study. The study was conducted at the same study site as the phase 3 trial, Samsung Medical Center (Seoul, Korea), from May 2018 to December 2022. Participants made weekly visits to the study site to receive idursulfase beta 0.5 mg/kg for 52 weeks and made an end-of-study visit at week 53. Idursulfase beta was delivered via intravenous infusion at a rate of 8 mL/hour for 15 min initially. If the infusion was tolerated well, the infusion rate was increased at a rate of 8 mL per hour every 15 min under the maximum rate of 100 mL per hour. The study was conducted according to the ethical principles of the Helsinki declaration and local regulations. The initial protocol and its subsequent amendments were approved by both the Ministry of Food and Drug Safety of Korea and the institutional review board of Samsung Medical Center.

### Study endpoints

The primary purpose of the study was to investigate the long-term safety of idursulfase beta. Safety outcomes evaluated as the primary endpoint included adverse events (AEs), acute AEs, adverse drug reactions (ADRs), serious adverse events (SAEs), immunogenicity, vital signs, physical examination, clinical laboratory tests, and 12-lead electrocardiography, which were assessed during two years of treatment. The severity of AE was graded according to the Common Terminology Criteria for Adverse Events (version 5.0). For safety analysis, AEs assessed as possibly, probably, or definitely related to the study drug were classified as ADRs. This conservative approach may have resulted in a higher reported ADR incidence. Among AEs occurring within 24 h after study drug infusion, the following were classified as acute AEs: anaphylaxis, respiratory distress, hypoxia, hypotension, urticaria, angioedema of throat or tongue, bronchospasm, cyanosis, erythema, edema, flushing, rash, vomiting, or wheezing. Immunogenicity tests included measurement of blood concentration of anti-idursulfase beta immunoglobulin G antibody (anti-drug antibody) and neutralizing antibody (NAb). If a positive result was confirmed for anti-drug antibody, analyzed using an electrochemiluminescence assay, two separate assays, a cell uptake NAb assay and an enzyme activity NAb assay, were carried out. Immunogenicity tests were performed every four months, and the blood samples were analyzed at Syneos Health (New Jersey, USA). Clinical laboratory tests and 12-lead electrocardiography were performed every four and six months, respectively, and the rest of the safety endpoints were assessed at each visit.

Efficacy endpoints, including 6-MWT, urine total GAG, urine heparan sulfate (HS), and urine dermatan sulfate (DS), were also analyzed for two years as secondary endpoints. 6-MWT was assessed by an independent, trained professional at a 6-month interval. Urine total GAG, HS, and DS were analyzed at the Global Clinical Central Lab (Gyeonggi-do, Korea) at every four-month interval. The urine concentration of total GAG was analyzed using a colorimetric dimethylmethylene blue assay with the Blyscan™ Sulfated Glycosaminoglycan Assay Kit (BioColor Ltd., Northern Ireland, UK). Urine HS and DS levels were quantified using the liquid chromatography tandem mass spectrometry.

### Statistical analysis

No formal sample size calculation was made due to the nature of the extension study. Individuals continually treated with idursulfase beta were classified as the maintenance group, whereas those who had received idursulfase during the phase 3 trial were classified as the switch group. Participants with missense variants were grouped as genotype 1 group, and the rest as genotype 2 group. Incidence of AEs was presented as percentages with 90% confidence intervals (CIs), along with the number of events. Continuous outcomes were summarized with descriptive statistics, and the significance of changes from baseline in each group was tested using the Wilcoxon signed-rank test, and the between-group difference was tested using the Wilcoxon rank-sum test.

An additional efficacy analysis using a mixed model for repeated measures (MMRM) was performed to test within-group changes and between-group differences. For analysis of absolute change from baseline, the model included baseline value and age as covariates, and treatment group and visits as fixed effects. For the analysis of percent change from baseline, the model included age as a covariate, with treatment group and visits as fixed effects. Least-square mean (LSM) changes from baseline and their 90% CIs were derived from the MMRM; results of the pre-planned analysis were reported in the text, while LSM changes with 90% CIs were plotted for visualization. Subgroup analysis evaluated percent changes in efficacy outcomes by neutralizing antibody development status or genotype group, and incidence of AE by neutralizing antibody development status within each genotype group. Any missing values of safety variables were not reconstituted. Given the ultra-rare nature of MPS II and the limited feasible sample size in a long-term extension study, a two-sided significance level of 10% was prespecified to balance type II error and clinical interpretability, consistent with prior rare disease trials. All statistical analyses were performed using Statistical Analysis Software (version 9.4; SAS Institute, Cary, NC, USA).

## Results

### Participants

**Fig. 1:**
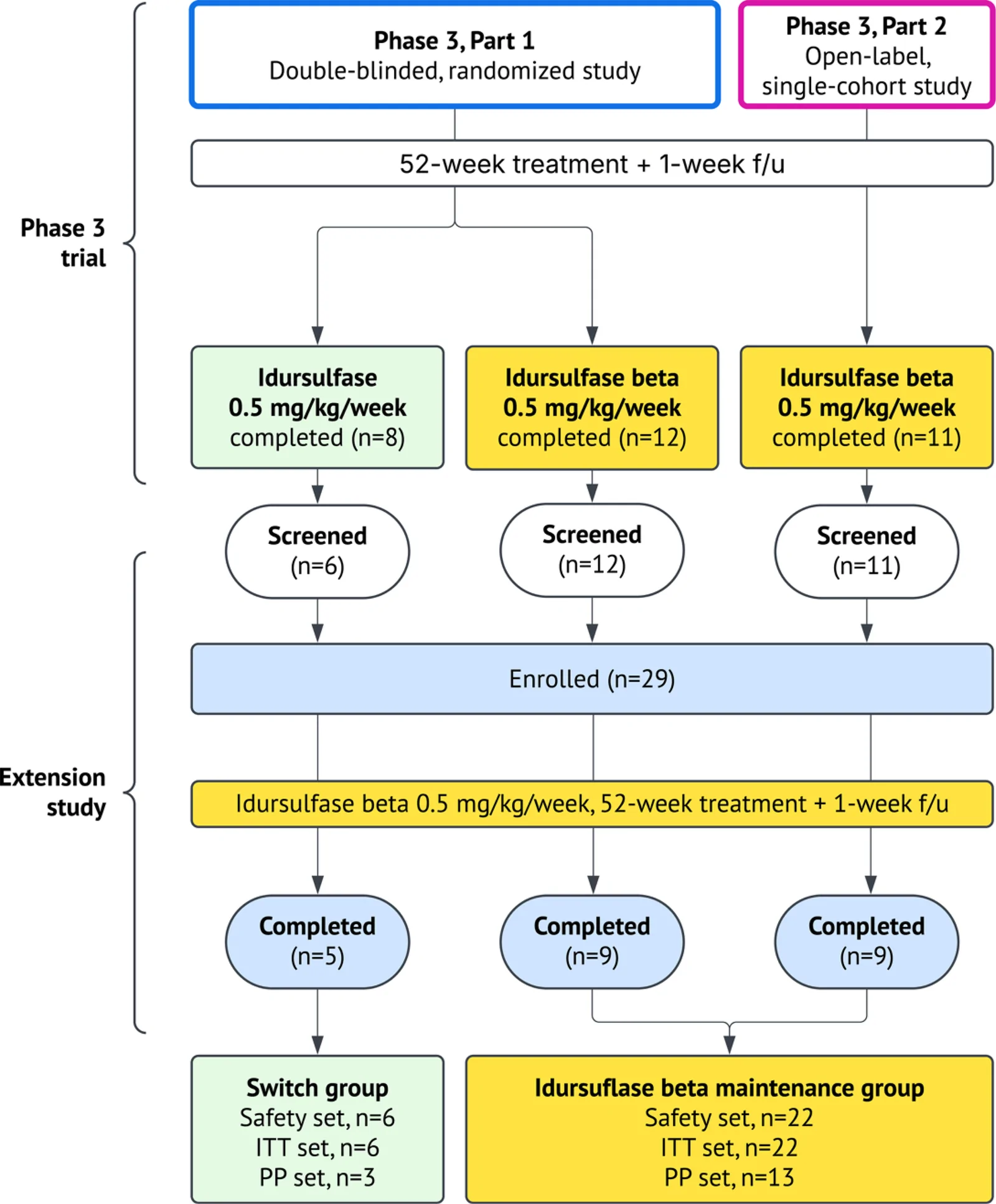
Patient disposition chart. One patient who had been treated with idursulfase beta outside the phase 3 trial was also enrolled in the extension study (not shown in the diagram); however, the patient’s data were excluded from the analysis due to an incomplete history of prior treatment. ITT, intention-to-treat; PP, per-protocol

Twenty-three participants who had received idursulfase beta either from Part 1 or Part 2 of the phase 3 trial were enrolled in the extension study; except for one participant who was lost to follow-up before the first administration of the drug in the extension study, the remaining 22 received idursulfase beta in the extension study (maintenance group). Meanwhile, six participants who had completed the double-blind, randomized part (Part 1) of the phase 3 trial were switched to the treatment with idursulfase beta in the extension study (switch group). One participant (13-year-old male Asian) who had been treated with idursulfase beta for over six months in a circumstance unrelated with the phase 3 trial was also enrolled in the extension study; however, the participant was not included in the analysis due to an incomplete history of previous treatment with idursulfase beta. The participant’s major study outcomes are summarized in the Tables S1 to S3. The remaining participants (22 in the maintenance group and 6 in the switch group) were included in the analysis (Fig. 1), and their demographic data and baseline characteristics are presented in Table 1. All participants were male and of Asian ethnicity, and the age ranged from 5 to 53 years in both groups, with the mean of 12.55 ± 9.99 years in the maintenance group and 20.67 ± 16.97 years in the switch group. Missense variants were the most common, with six patients (27.3%) in the maintenance group and three (50%) in the switch group. All participants had valvular heart disease and radiographic evidence of dysostosis multiplex; 92.9% had hepatosplenomegaly (21 in the maintenance group and 5 in the switch group) at baseline. A diverse range of medical histories characteristic of MPS II was identified at varying rates, such as inguinal or umbilical hernia (50.0%), adenoidal or tonsillar hypertrophy (32.1%), sleep apnea syndrome (7.1%), or otitis media (7.1%). Although some participants in this study cohort had severe MPS II, the study participants consisted solely of individuals who met the criteria for completing the 6-MWT. The mean total treatment period was 728.18 ± 47.26 days in the maintenance group. In the switch group, the mean total treatment period was 741.33 ± 41.92 days, which included 366.17 ± 1.17 days for idursulfase treatment and 356.33 ± 32.98 days for idursulfase beta treatment.

**Table 1:** Patient demographics and baseline characteristics

	Maintenance group (*n* = 22)	Switch group (*n* = 6)	Total (*N* = 28)
Age, yrs	12.55 ± 9.99	20.67 ± 16.97	14.29 ± 11.93
Male, n (%)	22 (100.0)	6 (100.0)	28 (100.0)
Ethnicity, n (%)			
Asian	22 (100.0)	6 (100.0)	28 (100.0)
Height, cm	122.51 ± 13.62	133.35 ± 12.87	124.83 ± 13.98
Weight, kg	31.20 ± 10.64	38.13 ± 11.90	32.68 ± 11.07
6-MWT, m	383.97 ± 94.42	371.82 ± 42.94	381.36 ± 85.45
6MWT Severity Score, n (%)			
Score 1 (≥ 500 m)	2 (9.1)	0 (0.0)	2 (7.1)
Score 2 (≥ 300 m to < 500 m)	18 (81.8)	6 (100.0)	24 (85.7)
Score 3 (< 300 m)	2 (9.1)	0 (0.0)	2 (7.1)
Urine GAG, mg/g Creatinine	344.00 ± 105.45	271.12 ± 120.58	328.38 ± 110.77
Genotype category, n (%)			
Complete gene deletion	0 (0.0)	0 (0.0)	0 (0.0)
Large gene rearrangement	1 (4.5)	0 (0.0)	1 (3.6)
Nonsense variants	6 (27.3)	0 (0.0)	6 (21.4)
Frameshift variants	2 (9.1)	2 (33.3)	4 (14.3)
Splice site variants	4 (18.2)	1 (16.7)	5 (17.9)
Missense variants	6 (27.3)	3 (50.0)	9 (32.1)
Unknown	0 (0.0)	0 (0.0)	0 (0.0)
Others	3 (13.6)	0 (0.0)	3 (10.7)
Genotype group, n (%)			
Genotype group 1^a^	6 (27.3)	3 (50.0)	9 (32.1)
Genotype group 2^b^	16 (72.7)	3 (50.0)	19 (67.9)

^a^Missense type. ^b^All other variants excluding missense variants

GAG, glycosaminoglycan; DS, dermatan sulfate; HS, heparan sulfate; 6-MWT, 6-minute walk test

### Safety

In the maintenance group, the incidence of AEs and the distribution of AE severity remained similar between the first and the second years of the treatment. Most of the AEs (96.3%, 156 out of 161 events across the groups) were classified as Grade 1 or 2; no AEs were Grade 4 or 5. During the two-year treatment period, the overall incidence rate of ADR was 63.6% (90% CI: 43.9%-80.4%) with a decreasing trend (54.5% and 13.6% in the first and second year, respectively) (Table 2). The incidence of all ADRs is presented in the Table S4. A total of five SAEs were reported in four participants (18.2%) in the maintenance group during the overall study period (Table S5). Acute AEs, detected in 31.8% (7/22) of the participants in the maintenance group, included rash (24 events in 5 participants), urticaria (32 events in three participants), and wheezing (one event in one participant). Although some participants experienced acute AEs repeatedly, all acute events were manageable with the modification of infusion or administration of medication (antihistamine or corticosteroid).

**Table 2:** Summary of adverse events

	Maintenance group (*n* = 22)	Switch group (*n* = 6)
**Total period (2 years)**		
AE	22 (100.0) [258]^a^	6 (100.0) [65]^a^
ADR	14 (63.6) [94]	6 (100.0) [37]
SAE	4 (18.2) [5]	4 (66.7) [6]
SADR	3 (13.6) [3]	4 (66.7) [6]
Acute AE	7 (31.8) [57]	1 (16.7) [2]
Withdrawal due to AE	0 (0) [0]	0 (0) [0]
**First year (Phase 3)**		
Treatment	Idursulfase beta	Idursulfase
AE	22 (100.0) [124]	6 (100.0) [48]
Grade 3	2 (9.1) [2]	1 (16.7) [1]
Keratitis^b, c^	0	1 (16.7) [1]
Splenic infarction^b, c^	1 (4.5) [1]	0
Rash^b, c^	1 (4.5) [1]	0
ADR	12 (54.6) [51]	6 (100.0) [30]
SAE	2 (9.1) [2]	2 (33.3) [4]
SADR	2 (9.1) [2]	2 (33.3) [4]
Acute AE	3 (13.6) [16]	1 (16.7) [2]
Withdrawal due to AE	0 (0) [0]	0 (0) [0]
**Second year (Extension)**		
Treatment	Idursulfase beta	Idursulfase beta
AE	19 (86.4) [133]	5 (83.3) [16]
Grade 3	1 (4.5) [3]	2 (33.3) [2]
Anal abscess^b, c^	0	1 (16.7) [1]
Tremor^b, c^	0	1 (16.7) [1]
Dental caries^d, e^	1 (4.5) [1]	0
Urticaria^b, c^	1 (4.5) [2]	0
ADR	3 (13.6) [43]	3 (50.0) [7]
SAE	2 (9.1) [3]	2 (33.3) [2]
SADR	1 (4.5) [1]	2 (33.3) [2]
Acute AE	4 (18.2) [41]	0 (0) [0]
Withdrawal due to AE	0 (0) [0]	0 (0) [0]

Data are number of patients (%) with the number of events in the square brackets. ^a^In both groups, one adverse event that occurred between the last visit of the phase 3 trial and the first visit of the extension study was reflected in the total number of AE; however, the event was not reflected in either of the periods. ^b^Assessed as an ADR. ^c^Resolved during the study period. ^d^Not related to idursulfase beta. ^e^Ongoing at the end of the study

ADR, adverse drug reaction; AE, adverse event; SADR, serious adverse drug reaction; SAE, serious adverse event

In the switch group, the six participants (100.0%) and five participants (83.3%) experienced AEs during the first and second year, respectively, with the decreasing trend in the occurrence of ADRs over time (100.0% during the first year and 50.0% during the second year) (Table 2). Two events of urticaria, recorded as acute AEs, occurred in one participant while receiving idursulfase; however, none of the participants in the switch group had acute AEs during the second year.

None were withdrawn due to any AEs; no death was reported during the two-year study period. No clinically relevant safety signals were detected in clinical laboratory tests, physical exams, vital signs, or 12-lead electrocardiography.

### Six-minute walk test

**Fig. 2:**
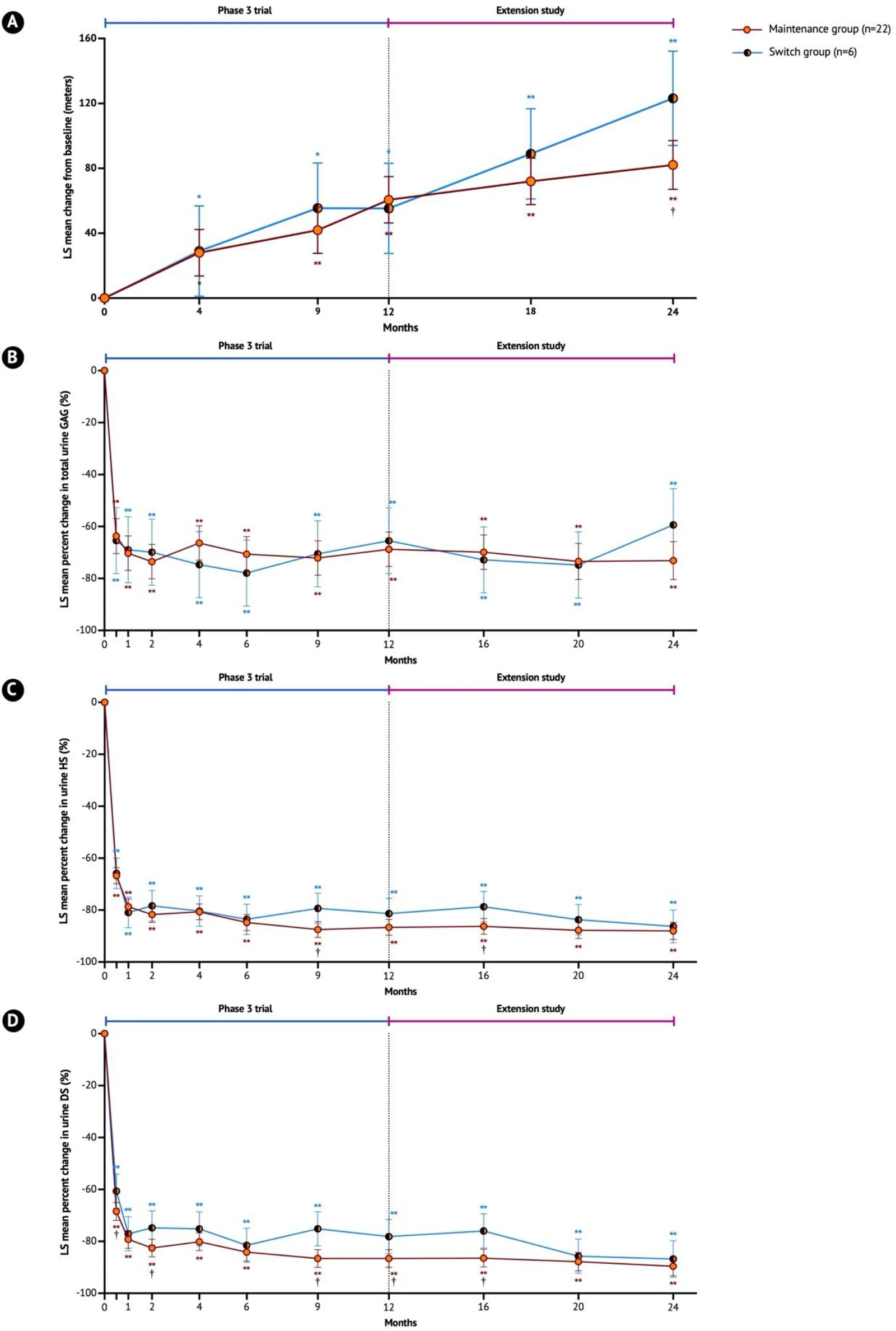
Least-square (LS) mean change or percent change from baseline in efficacy parameters. (**A**) 6-minute walk test (6-MWT) distance. (**B**) Urine total glycosaminoglycans (GAG). (**C**) Urine heparan sulfate (HS). (**D**) Urine dermatan sulfate (DS). The error bars represent 90% confidence intervals for least-square means. ^*^Significant change from baseline at *p* < 0.05; ^**^Significant change from baseline at *p* < 0.0001. ^†^Significant between-group difference at *p* < 0.1

At baseline of the phase 3 trial, mean 6-MWT was 383.97 ± 94.42 m in the maintenance group and 371.82 ± 42.94 m in the switch group. A significant increase in 6-MWT was observed after four months of treatment in both groups; the increasing trend in the distance measured in 6-MWT continued till the end of the extension study (Fig. 2A). LSM change in 6-MWT from baseline to Month 24 was 82.08 ± 99.07 m (90% CI: 67.07–97.08; 23.1% increase, *p* < 0.0001) in the maintenance group and 123.11 ± 17.55 m (90% CI: 94.07-152.15; 33.2% increase, *p* < 0.0001) in the switch group. The between-group difference in change in 6-MWT was statistically significant only at Month 24 (*p* = 0.0412 for absolute change, *p* = 0.0981 for percent change), whereas it was not significant at the prior visits (*p* > 0.1 for both absolute and percent changes) (Tables S6 and S7). When reviewing the individual data, all but one participant in the maintenance group improved on the 6-MWT outcome; no clear relationship was observable between age or baseline 6-MWT distance (Fig. S1).

### Urinary GAG

A significant reduction from baseline in urine total GAG was observed from one month after treatment, and the level of reduction was maintained during the whole study period (Fig. 2B). LSM from baseline to Month 24 was − 247.50 ± 13.97 mg/g creatinine (73.1% reduction, *p* < 0.0001) in the maintenance group and − 210.18 ± 26.66 mg/g creatinine (59.4% reduction, *p* < 0.0001) in the switch group. The between-group differences in the absolute and percent change in urine total GAG were not statistically significant at Month 24 (*p* = 0.2174 and *p* = 0.1502, respectively) (Tables S8 and S9, Fig. 2B). The changes in urine HS and DS were in line with those of urine total GAG (Fig. 2C and D). At Month 24, the percent reduction in urine DS from baseline amounted to 89.6% (*p* < 0.0001) in the maintenance group and 86.8% (*p* < 0.0001) in the switch group. As for urine HS, percent reduction from baseline at Month 24 was 88.0% (*p* < 0.0001) and 86.3% (*p* < 0.0001), respectively. No significant between-group difference was detected at Month 24 (Tables S10 to S13).

### Immunogenicity

**Fig. 3:**
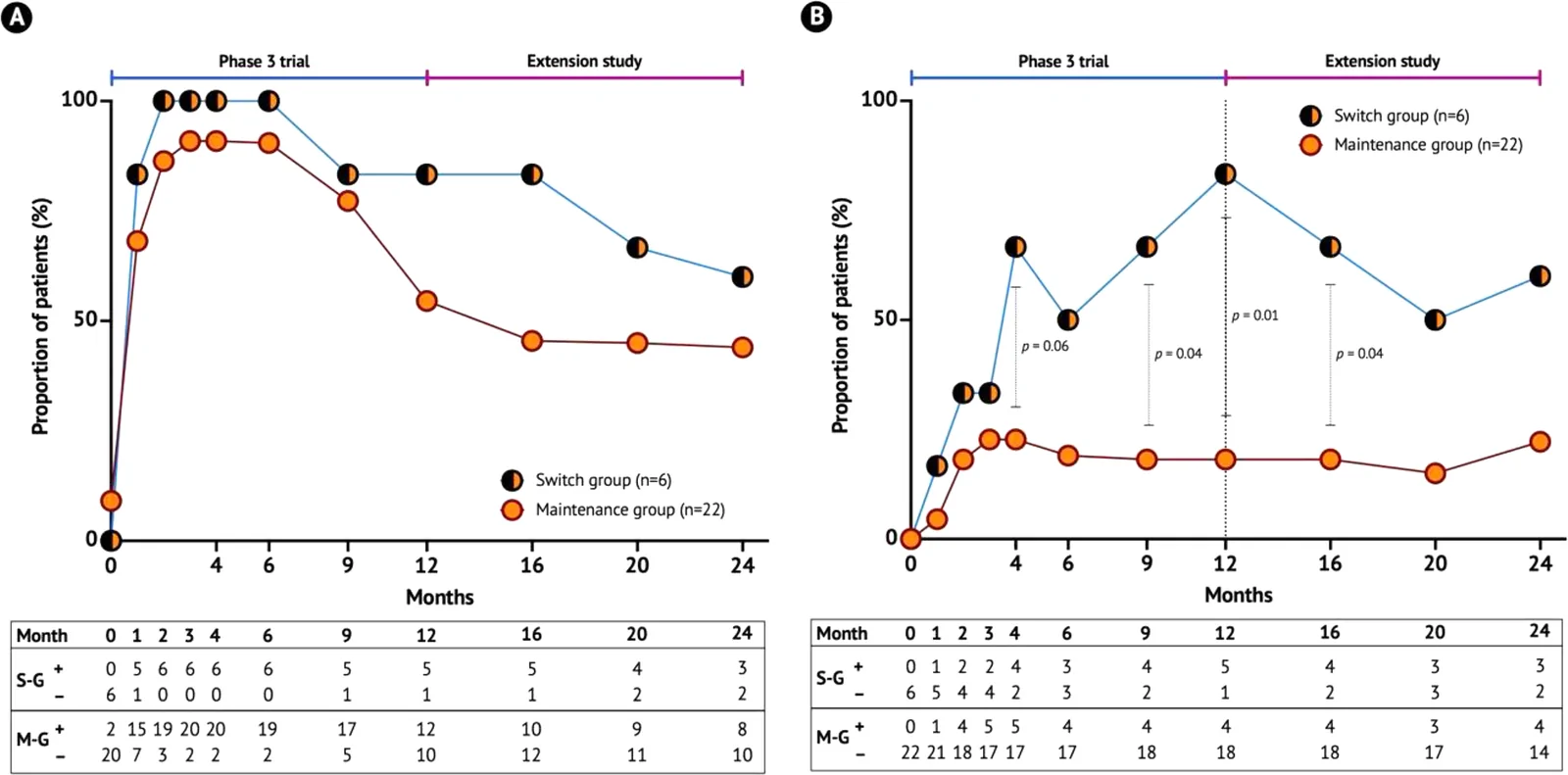
Proportion of patients with positive anti-drug antibody (**A**) and positive neutralizing antibody (**B**). Neutralizing antibody tests were performed only for those with positive anti-drug antibody using two methods, the cell uptake neutralizing assay and the enzyme activity neutralizing assay. If either of the two assay results was positive, the patient was counted as one with positive neutralizing antibody. *P*-values from Fisher’s exact test are shown on the graph if a significant between-group difference was detected. The table below each figure presents the actual number of participants who had positive (+) or negative (–) outcomes of the test at each timepoint. M-G: maintenance group; S-G: switch group. Month 0 was the baseline

Anti-drug antibody was detected in most of the participants early in the treatment period (Fig. 3A). However, the proportion of participants developing anti-drug antibody began to decrease after six months of treatment, and it remained between 36.4% and 54.5% in the maintenance group during the extension period. The proportion of participants with positive anti-drug antibody in the switch group also began to decrease from Month 6, reaching 50.0% at the end of the extension study, driven by a sustained decline. Not all participants with positive anti-drug antibody had neutralizing antibody. In the maintenance group, the proportion of individuals tested positively to either of the two neutralizing antibody assays ranged from 4.5% to 22.7% during the two-year period (Fig. 3B). The individual immunogenicity results visualized with timepoints are provided in Fig. S2.

### Safety and efficacy assessments by neutralizing antibody status and genotype group

Subgroup analysis showed that the incidence of overall AE did not differ by neutralizing antibody development status; however, the incidence of SAE or acute AE was higher in the subgroup with at least one positive neutralizing antibody result (Table S14). Meanwhile, the incidence of acute adverse reactions was relatively high in genotype 2 (carriers of non-missense mutations) who tested positive for neutralizing antibodies (46 adverse reactions occurred in 6 of the 11 participants).

The subgroup analysis of efficacy outcomes (6-MWT and urine total GAG) showed that the neutralizing antibody development didn’t affect the treatment outcomes (Table S15) as the percent change in 6-MWT was significantly greater in the neutralizing positive group than in the negative group (29.1% vs. 16.3%, *p* = 0.0087). Efficacy outcomes also consistently improved after 24 months, regardless of genotype (Table S16).

## Discussion

To our knowledge, this is the first study to report two-year outcomes including a cohort switched from idursulfase to idursulfase beta. This study includes the 52-week outcomes of the treatment group that switched from idursulfase to idursulfase beta. In this open-label extension study, the safety profile of idursulfase beta after two years of treatment was similar to that after one year, yet with the lower incidence of ADRs. The proportion of participants with anti-drug antibody tended to decrease starting at six months of the treatment; neutralizing antibodies remained relatively low during the whole study period (4.5–22.7%).

The safety outcomes observed in the switch group during the extension period were also noteworthy. Switching to idursulfase beta did not boost an additional synthesis of anti-drug antibodies, which implies that the immunogenicity profile of the idursulfase beta is likely favorable. The positive treatment effect on the efficacy outcomes, the 6-MWT distance, and urine GAG level, was maintained consistently with treatment duration, and no meaningful difference in either parameter was observed between the maintenance and switch groups at the end of the study period. All the findings mentioned above point to idursulfase beta as a safe and efficacious treatment option for long-term management of patients with MPS II, not only as a first-line therapy but also as an alternative for therapy switching.

Overall, the safety profile of idursulfase beta documented in the present study appeared to be similar to those of idursulfase and idursulfase beta reported previously [14–16, 20, 21], with most AEs being mild or moderate in severity. While the frequency of AEs remained generally stable throughout the study period, the incidence of ADRs decreased substantially between the first and second years of treatment, from 54.5% to 13.6% in the maintenance group, and from 100.0% to 50.0% in the switch group. This implies that idursulfase beta would be a safe, long-term treatment option for patients with MPS II, and some patients might even develop better tolerance of the drug with its prolonged administration. While some participants repeatedly experienced acute AEs, these events were managed by adjusting the infusion rate and by pharmacological intervention (antihistamines or corticosteroids). Individual differences in the sensitivity to idursulfase beta might be related to MPS II genotype. Indeed, the participants who experienced repeated acute AEs in this study had genotypes other than a missense variant. Likewise, both a recent phase 3 trial of idursulfase beta and a phase 2/3 study of idursulfase showed that participants with missense variants were less prone to SAEs and acute infusion-related AEs than those with other genotype types [20, 21, 24].

While most participants presented with anti-drug antibody early in the study, the proportion of positive rate started to decrease after six months from the initial dosing, and at the end of the second year, it reached 36.4% in the maintenance group and 50.0% in the switch group. Furthermore, not all participants with anti-drug antibodies developed neutralizing antibodies. Less than 20% of the participants in the maintenance group had positive neutralizing antibodies during the extension period, similar to a prior study of idursulfase, where neutralizing antibodies were detected in 22.3% (19/85) of participants after 105 weeks [16]. In addition, the proportion of patients with neutralizing antibodies observed in the switch group presented herein also implies that switching from idursulfase to idursulfase beta did not impact patients’ immune responses against the ERT, showing a decreased sensitivity over time after switching. Meanwhile, experiences with ERT in other lysosomal storage diseases suggest that some recombinant enzymes sharing the same amino acid sequence, e.g., agalsidase alpha and beta used in the treatment of Fabry disease, may differ in terms of their immunogenicity [25].

As suggested in the study of idursulfase [24], our study results also indicate that the presence of neutralizing antibodies against idursulfase beta does not necessarily affect treatment outcomes negatively, which indeed was supported by a substantial improvement in the efficacy outcomes over a 24-month period of the present study. It was also noted that the presence of neutralizing antibodies did not significantly affect the results of 6-MWT and urine GAG levels in a recent phase 3 study of idursulfase beta [20]. Taken together, longer duration of idursulfase beta treatment, as a sole treatment and as a prolonged treatment for switching from idursulfase as well, does not appear to be associated with decreased pharmacological activity of the drug. As such, the subgroup analysis of this study’s results suggests that the efficacy outcomes were consistently improved, regardless of the genotype. Although several factors, including the study design and number of patients analyzed, limit assessing this aspect, there seems to be no apparent association between genotypes and the efficacy of ERTs for MPS II in this study. Of note, it was once emphasized that genotype and antibody status have limited predictive value for individual patient outcomes [26].

At the end of the present study, the 6-MWT results for the maintenance group and the switch group showed significant improvement compared to baseline, with increases of 23.1% and 33.2%, respectively; these figures are higher than the 18.2% increase in 6-MWT distance recorded after one year in a recent Phase 3 clinical trial of idursulfase beta [20]. These observations suggest that patients with MPS II might maintain treatment benefit from switching from idursulfase to idursulfase beta. The change in the 6-MWT distance (82.08 m, 23.1% increase) observed in this study was also greater than in a two-year phase 2/3 study of idursulfase (42 m, 11.7% increase) or other long-term studies of idursulfase (31.8 m to 67 m increase during three to ten year follow-up) [16, 27–29], and all but one participant achieved improvement from baseline in the 6-MWT distance. While further research is warranted on the issue, as it is well known that the result of 6-MWT reflects the function of at least three body systems, respiratory, cardiovascular, and musculoskeletal [30, 31], the substantial improvement in this parameter documented herein implies that prolonged treatment with idursulfase beta might exert a clinically meaningful improvement on cardiorespiratory performance and mobility in patients with MPS II. Although direct cross-study comparisons should be interpreted with caution due to differences in study design and patient populations, the magnitude of improvement observed in the present study appears clinically meaningful.

Meanwhile, it should be noted that the main phase 3 trial was primarily designed to evaluate the effect of IV ERT on somatic manifestations and systemic functional outcomes in patients with MPS II who were able to complete 6-MWT, even if they exhibit a neuropathic phenotype. Consistent with this objective, patients aged 5 years or older who were able to perform the 6-MWT were enrolled, while those unable to perform the test were excluded at screening. Therefore, patients with a severe neuronopathic phenotype may have been underrepresented in the study population, and the current study has limited ability to assess outcomes in patients with substantial neurological involvement. This design is also in line with the current understanding that IV ERT has limited evidence for improving CNS-related manifestations. Thus, relatively more ambulatory young patients who were less likely to represent the severe neuronopathic phenotype could have performed better with increasing age, which might have positively impacted the 6-MWT performance. However, the individual data revealed no obvious indication of favorable outcomes in younger participants or those with greater 6-MWT performance at baseline.

A significant decrease in urine total GAG was documented after 24 months of the present study, by 73.1% and 59.4% of the baseline value in the maintenance and switch groups, respectively. The changes in urine DS and HS followed a similar pattern, with 89.6% and 86.8% decreases in DS and an 88.0% and 86.3% reductions in HS in the maintenance and switch groups, respectively, at Month 24. The relative change in urine total GAG was slightly greater than in the recent phase 3 trial of idursulfase beta,^20^ which implies that the drug might at least maintain its therapeutic effectiveness with prolonged treatment. Additionally, no statistically significant differences in the relative change in urine total GAG were found between the maintenance and switch groups included in the present study, with the reduction rate similar to that observed in a previous long-term study of idursulfase [29]. These findings, along with the sustained improvement in the 6-MWT distance gain between maintenance and switch groups, imply that switching from idursulfase to prolonged treatment with idursulfase beta may help maintain therapeutic benefit after prior idursulfase treatment and may represent a clinically viable alternative following idursulfase treatment.

This study has several limitations. The open-label design and the small sample size, particularly in the switch group, limit the generalizability of the findings. The lack of reporting on pulmonary functions, organ volumes, or cardiac outcomes limits the interpretation of the full clinical impact of idursulfase beta. In addition, the absence of a concurrent control group precludes definitive comparative conclusions. Nevertheless, given the rarity of MPS II, these long-term extension data provide clinically valuable insights into sustained treatment and switching strategies.

## Conclusions

To summarize, the results of the present study suggest that idursulfase beta is an efficacious and safe option for the treatment of MPS II. Pharmacological activity of the drug during a prolonged, two-year treatment did not seem to be limited by its immunogenicity, and therapeutic outcomes were at least as good as at early treatment phases. Similarly satisfactory were the outcomes in patients switched from idursulfase to idursulfase beta. Thus, idursulfase beta may serve as an effective alternative to idursulfase in the management of MPS II. The results of the present study and yet unpublished findings of a recently completed long-term observational study shall ultimately confirm whether idursulfase beta is also suitable for prolonged treatment of MPS II. 

## Supplementary Information

Below is the link to the electronic supplementary material.

Supplementary Material 1: The following supplementary information can be found online. Tables S1 to S3: Safety, efficacy, and immunogenicity results in the one patient enrolled only in the extension study. Table S4: Incidence of adverse drug reactions. Table S5: Serious adverse events reported during the whole study period. Table S6: Observed mean at each visit and absolute change from baseline in 6-MWT. Table S7: Percent change from baseline in 6-MWT. Table S8: Observed mean at each visit and absolute change from baseline in urine total GAG. Table S9: Percent change from baseline in urine total GAG. Table S10: Observed mean at each visit and absolute change from baseline in urine dermatan sulfate. Table S11: Percent change from baseline in urine dermatan sulfate. Table S12: Observed mean at each visit and absolute change from baseline in urine heparan sulfate. Table S13: Percent change from baseline in urine heparan sulfate. Table S14: Incidence of adverse events by neutralizing antibody formation status and genotype type. Table S15: Percent changes at Month 24 from baseline in efficacy outcomes by neutralizing antibody development status. Table S16: Percent changes at Month 24 from baseline in efficacy outcomes by genotype. Fig. S1 Visualization of individual patient’s percent change in 6-minute walk test. Fig. S2 Visualization of individual patient’s immunogenicity results.

## Data Availability

The datasets generated and/or analyzed during the current study are available from the corresponding author on reasonable request.

## Abbreviations

ADR: Adverse drug reactions
AE: Adverse events
CI: Confidence intervals
DS: Dermatan sulfate
ERT: Enzyme replacement therapy
HS: Heparan sulfate
I2S: Iduronate-2-sulfatase
IV: Intravenously
LSM: Least-square mean
MMRM: Mixed model for repeated measures
NAb: Neutralizing antibody
SAE: Serious adverse events
6-MWT: Six-minute walk test
