# Supplementary material for: Sustained clinical benefit of idursulfase beta in mucopolysaccharidosis II: two-year experience from a phase 3 extension study including patients switched from idursulfase
Source: Orphanet J Rare Dis. 2026 Jul 9;21:343. doi: 10.1186/s13023-026-04492-2 (PMC13637351; doi:10.1186/s13023-026-04492-2)
Supplement: Supplementary file 1 [file 13023_2026_4492_MOESM1_ESM.docx]

**Supplementary information**

**Table S1** List of adverse events experienced by one patient enrolled only in the extension study

|  | **Grade 1** | **Grade 2** | **Grade 3** | **Grade 4 or 5** |
| --- | --- | --- | --- | --- |
| **No. of events** | **5** | **6** | **1** | **0** |
| Bronchitis | 0 | 1 | 0 | 0 |
| Erythema | 1 | 0 | 0 | 0 |
| Toothache | 0 | 1 | 0 | 0 |
| Productive cough | 0 | 1 | 0 | 0 |
| Swelling face | 1 | 0 | 0 | 0 |
| Gait disturbance | 1 | 0 | 0 | 0 |
| Mass | 1 | 0 | 0 | 0 |
| Skin laceration | 0 | 1 | 0 | 0 |
| Deafness | 0 | 0 | 1 | 0 |
| Epilepsy | 0 | 1 | 0 | 0 |
| Hepatomegaly | 1 | 0 | 0 | 0 |
| Insomnia | 0 | 1 | 0 | 0 |

The patient’s exposure to idursulfase beta during the extension study was 395 days and the treatment compliance was 98.07%.

**Table S2** Changes in efficacy endpoints in one patient enrolled only in the extension study

|  | **Baseline** | **Month 16** | **Month 18** | **Month 20** | **Month 24** |
| --- | --- | --- | --- | --- | --- |
| 6-MWT, meter | 234.40 | NA | 324.60 | NA | 240.00 |
| Change from baseline | 0 | NA | 90.20 | NA | 5.60 |
| Urine total GAG, mg/g Creatinine | 345.80 | 63.30 | NA | 55.79 | 61.10 |
| Change from baseline | 0 | -282.50 | NA | -290.01 | -284.70 |
| Urine DS, mg/g Creatinine | 231.60 | 14.30 | NA | 13.95 | 15.60 |
| Change from baseline | 0 | -217.30 | NA | -217.65 | -216.00 |
| Urine HS, mg/g Creatinine | 651.60 | 50.40 | NA | 41.84 | 44.10 |
| Change from baseline | 0 | -601.20 | NA | -609.76 | -607.50 |

GAG, glycosaminoglycan; DS, dermatan sulfate; HS, heparan sulfate; 6-MWT, 6-minute walk test.

**Table S3** Immunogenicity assay results in one patient enrolled only in the extension study

|  | **Baseline** | **Month 16** | **Month 20** | **Month 24** |
| --- | --- | --- | --- | --- |
| ADA | positive | positive | negative | negative |
| Cell uptake neutralizing assay | negative | positive | not done | not done |
| Enzyme activity neutralizing assay | negative | negative | not done | not done |

ADA, anti-drug antibody.

**Table S4** Incidence of adverse drug reactions

|  | **Switch group**  **(n=6)** | | **Maintenance group**  **(n=22)** |
| --- | --- | --- | --- |
|  | **First year with**  **idursulfase** | **Second year with idursulfase beta** | **Two years with**  **idursulfase beta** |
| **Any ADR** | **6 (100.0) [30]** | **3 (50.0) [7]** | **14 (63.6) [94]** |
| **Cardiac disorders** | **5 (83.3) [7]** | **1 (16.7) [1]** | **4 (18.2) [4]** |
| Cardiac valve disease | 5 (83.3) [5] | 0 | 3 (13.6) [3] |
| Bundle branch block right | 1 (16.7) [1] | 0 | 0 |
| Palpitations | 0 | 1 (16.7) [1] | 0 |
| Right atrial enlargement | 0 | 0 | 1 (4.5) [1] |
| Ventricular extrasystoles | 1 (16.7) [1] | 0 | 0 |
| **Infections and infestations** | **3 (50.0) [5]** | **2 (33.3) [2]** | **6 (27.3) [7]** |
| Anal abscess | 0 | 1 (16.7) [1] | 0 |
| Conjunctivitis | 1 (16.7) [1] | 0 | 0 |
| Folliculitis | 1 (16.7) [1] | 0 | 0 |
| Hordeolum | 1 (16.7) [1] | 0 | 0 |
| Laryngopharyngitis | 1 (16.7) [1] | 0 | 0 |
| Laryngitis | 0 | 0 | 1 (4.5) [1] |
| Nasopharyngitis | 1 (16.7) [1] | 0 | 5 (22.7) [5] |
| Otitis externa | 0 | 1 (16.7) [1] | 0 |
| Otitis media chronic | 0 | 0 | 1 (4.5) [1] |
| **Eye disorders** | **2 (33.3) [4]** | **1 (16.7) [1]** | **1 (4.5) [1]** |
| Eye pain | 0 | 1 (16.7) [1] | 0 |
| Keratitis | 1 (16.7) [1] | 0 | 0 |
| Macular oedema | 1 (16.7) [1] | 0 | 0 |
| Maculopathy | 1 (16.7) [1] | 0 | 0 |
| Optic neuropathy | 1 (16.7) [1] | 0 | 0 |
| Panophthalmitis | 0 | 0 | 1 (4.5) [1] |
| **Renal and urinary disorders** | **1 (16.7) [1]** | **1 (16.7) [1]** | **1 (4.5) [1]** |
| Renal cyst | 1 (16.7) [1] | 1 (16.7) [1] | 0 |
| Haematuria | 0 | 0 | 1 (4.5) [1] |
| **Respiratory, thoracic and mediastinal disorders** | **2 (33.3) [4]** | **0** | **3 (13.6) [4]** |
| Nasal pruritus | 1 (16.7) [1] | 0 | 0 |
| Productive cough | 0 | 0 | 2 (9.1) [2] |
| Respiratory disorder | 1 (16.7) [2] | 0 | 1 (4.5) [1] |
| Cough | 0 | 0 | 1 (4.5) [1] |
| Sneezing | 1 (16.7) [1] | 0 | 0 |
| **Blood and lymphatic system disorders** | **1 (16.7) [2]** | **0** | **2 (9.1) [2]** |
| Anaemia | 1 (16.7) [1] | 0 | 0 |
| Poikilocytosis | 1 (16.7) [1] | 0 | 0 |
| Splenic infarction | 0 | 0 | 1 (4.5) [1] |
| Thrombocytopenia | 0 | 0 | 1 (4.5) [1] |
| **Gastrointestinal disorders** | **1 (16.7) [1]** | **0** | **2 (9.1) [5]** |
| Abdominal pain | 0 | 0 | 1 (4.5) [1] |
| Diarrhoea | 0 | 0 | 1 (4.5) [2] |
| Gastritis | 1 (16.7) [1] | 0 | 0 |
| Lip swelling | 0 | 0 | 1 (4.5) [1] |
| Mouth ulceration | 0 | 0 | 1 (4.5) [1] |
| **General disorders and administration site conditions** | **0** | **1 (16.7) [1]** | **1 (4.5) [2]** |
| Chest discomfort | 0 | 1 (16.7) [1] | 0 |
| Peripheral swelling | 0 | 0 | 1 (4.5) [2] |
| **Hepatobiliary disorders** | **1 (16.7) [1]** | **0** | **0** |
| Hepatic lesion | 1 (16.7) [1] | 0 | 0 |
| **Musculoskeletal and connective tissue disorders** | **1 (16.7) [2]** | **0** | **2 (9.1) [2]** |
| Arthralgia | 0 | 0 | 1 (4.5) [1] |
| Back pain | 1 (16.7) [1] | 0 | 0 |
| Flank pain | 1 (16.7) [1] | 0 | 0 |
| Joint swelling | 0 | 0 | 1 (4.5) [1] |
| **Nervous system disorders** | **0** | **1 (16.7) [1]** | **1 (4.5) [1]** |
| Tonic convulsion | 0 | 0 | 1 (4.5) [1] |
| Tremor | 0 | 1 (16.7) [1] | 0 |
| **Investigations** | **0** | **0** | **2 (9.1) [2]** |
| Ejection fraction decreased | 0 | 0 | 1 (4.5) [1] |
| Electrocardiogram low voltage | 0 | 0 | 1 (4.5) [1] |
| **Injury, poisoning and procedural complications** | **0** | **0** | **1 (4.5) [1]** |
| Foreign body in ear | 0 | 0 | 1 (4.5) [1] |
| **Skin and subcutaneous tissue disorders** | **1 (16.7) [1]** | **0** | **2 (9.1) [7]** |
| Pruritus | 0 | 0 | 2 (9.1) [6] |
| Angioedema | 0 | 0 | 1 (4.5) [1] |
| Mechanical urticaria | 1 (16.7) [1] | 0 | 0 |
| **Acute Adverse Event** | **1 (16.7) [2]** | **0** | **6 (27.3) [55]** |
| Rash | 0 | 0 | 5 (22.7) [23] |
| Urticaria | 1 (16.7) [2] | 0 | 3 (13.6) [32] |

**Table S5** Serious adverse events reported during the whole study period

|  | **Switch group**  **(n=6)** | **Maintenance group**  **(n=22)** |
| --- | --- | --- |
| **SAE** | **4 (66.7) [6]** | **4 (18.2) [5]** |
| Keratitis^a,b^ | 1 (16.7) [1] | 0 (0) [0] |
| Macular oedema^a,c^ | 1 (16.7) [1] | 0 (0) [0] |
| Maculopathy^a,c^ | 1 (16.7) [1] | 0 (0) [0] |
| Optic neuropathy^a,c^ | 1 (16.7) [1] | 0 (0) [0] |
| Inguinal hernia^b^ | 0 (0) [0] | 1 (4.5) [1] |
| Umbilical hernia^b^ | 0 (0) [0] | 1 (4.5) [1] |
| Splenic infarction^a,b^ | 0 (0) [0] | 1 (4.5) [1] |
| Anal abscess^a,b^ | 1 (16.7) [1] | 0 (0) [0] |
| Tremor^a,b^ | 1 (16.7) [1] | 0 (0) [0] |
| Rash^a,b^ | 0 (0) [0] | 1 (4.5) [1] |
| Urticaria^a,b^ | 0 (0) [0] | 1 (4.5) [1] |

^a^Assessed as a serious adverse drug reaction. ^b^Resolved during the study period. ^c^Occurred during the phase 3 trial period and remained in stable conditions throughout the extension study. All serious adverse events (SAEs) were in grade 2 or 3 in severity; no adverse events, including SAEs, caused early withdrawal.

**Table S6** Observed mean at each visit and absolute change from baseline in 6-MWT

| **Unit: meters** |  | **Switch**  **(n=6)** | **Maintenance**  **(n=22)** |
| --- | --- | --- | --- |
| Baseline | Mean±SD | 371.82±42.94 | 383.97±94.42 |
| Month 4 | Mean±SD | 398.00±67.66 | 412.77±90.99 |
| Change from baseline | LS mean±SE | 29.00±16.80 | 27.96±8.65 |
|  | 90% CI for LS mean | (1.20, 56.79) | (13.64, 42.28) |
|  | *p-*value^a^ | 0.0863 | 0.0015 |
|  | *p-*value^b^ | 0.9566 | |
| Month 9 | Mean±SD | 424.50±76.12 | 426.71±101.98 |
| Change from baseline | LS mean±SE | 55.50±16.80 | 41.90±8.65 |
|  | 90% CI for LS mean | (27.70, 83.29) | (27.58, 56.22) |
|  | *p-*value^a^ | 0.0012 | <0.0001 |
|  | *p-*value^b^ | 0.4772 | |
| Month 12^c^ | Mean±SD | 424.32±70.77 | 445.44±98.05 |
| Change from baseline | LS mean±SE | 55.31±16.80 | 60.62±8.65 |
|  | 90% CI for LS mean | (27.51, 83.11) | (46.30, 74.94) |
|  | *p-*value^a^ | 0.0012 | <0.0001 |
|  | *p-*value^b^ | 0.7812 | |
| Month 18 | Mean±SD | 457.92±67.78 | 456.80±95.46 |
| Change from baseline | LS mean±SE | 88.91±16.80 | 71.99±8.65 |
|  | 90% CI for LS mean | (61.11, 116.71) | (57.67, 86.31) |
|  | *p-*value^a^ | <0.0001 | <0.0001 |
|  | *p-*value^b^ | 0.3766 | |
| Month 24 | Mean±SD | 481.00±78.84 | 463.14±102.48 |
| Change from baseline | LS mean±SE | 123.11±17.55 | 82.08±9.07 |
|  | 90% CI for LS mean | (94.07, 152.15) | (67.07, 97.08) |
|  | *p-*value^a^ | <0.0001 | <0.0001 |
|  | *p-*value^b^ | 0.0412 | |

^a^*p-*value calculated from the mixed-model for repeated measure testing within-group change from baseline. ^b^*p-*value calculated from the mixed-model for repeated measure testing between-group change from baseline. ^c^Baseline measurements at the extension study were treated as values at Month 12.

6-MWT, 6-minute walk test; CI, confidence interval; LS, least-square; SD, standard deviation; SE, standard error of mean.

**Table S7** Percent change from baseline in 6-MWT

| **Unit: %** |  | **Switch**  **(n=6)** | **Maintenance**  **(n=22)** |
| --- | --- | --- | --- |
| Month 4 |  |  |  |
| Change from baseline | LS mean±SE | 7.59±5.13 | 7.93±2.64 |
|  | 90% CI for LS mean | (-0.91, 16.08) | (3.55, 12.30) |
|  | *p-*value^a^ | 0.1415 | 0.0032 |
|  | *p-*value^b^ | 0.9534 | |
| Month 9 |  |  |  |
| Change from baseline | LS mean±SE | 14.92±5.13 | 10.81±2.64 |
|  | 90% CI for LS mean | (6.43, 23.42) | (6.44, 15.19) |
|  | *p-*value^a^ | 0.0042 | <0.0001 |
|  | *p-*value^b^ | 0.4822 | |
| Month 12^c^ |  |  |  |
| Change from baseline | LS mean±SE | 15.07±5.13 | 16.42±2.64 |
|  | 90% CI for LS mean | (6.58, 23.57) | (12.05, 20.80) |
|  | *p-*value^a^ | 0.0038 | <0.0001 |
|  | *p-*value^b^ | 0.8169 | |
| Month 18 |  |  |  |
| Change from baseline | LS mean±SE | 24.00±5.13 | 19.98±2.64 |
|  | 90% CI for LS mean | (15.51, 32.50) | (15.60, 24.35) |
|  | *p-*value^a^ | <0.0001 | <0.0001 |
|  | *p-*value^b^ | 0.4914 | |
| Month 24 |  |  |  |
| Change from baseline | LS mean±SE | 33.18±5.35 | 23.07±2.76 |
|  | 90% CI for LS mean | (24.33, 42.03) | (18.50, 27.64) |
|  | *p-*value^a^ | <0.0001 | <0.0001 |
|  | *p-*value^b^ | 0.0981 | |

^a^*p-*value calculated from the mixed-model for repeated measure testing within-group change from baseline. ^b^*p-*value calculated from the mixed-model for repeated measure testing between-group change from baseline. ^c^Baseline measurements at the extension study were treated as values at Month 12.

6-MWT, 6-minute walk test; CI, confidence interval; LS, least-square; SD, standard deviation; SE, standard error of mean.

**Table S8** Observed mean at each visit and absolute change from baseline in urine total GAG

| **Unit: mg GAG/g Creatinine** | | **Switch**  **(n=6)** | **Maintenance**  **(n=22)** |
| --- | --- | --- | --- |
| Baseline | Mean±SD | 271.12±120.58 | 344.00±105.45 |
| Week 2 | Mean±SD | 100.09±44.95 | 118.47±41.44 |
| Change from baseline | LS mean±SE | -218.19±24.33 | -212.50±12.94 |
|  | 90% CI for LS mean | (-258.35, -178.02) | (-233.87, -191.14) |
|  | *p-*value^a^ | <0.0001 | <0.0001 |
|  | *p-*value^b^ | 0.8372 | |
| Month 1 | Mean±SD | 88.45±37.35 | 97.34±49.12 |
| Change from baseline | LS mean±SE | -229.82±24.33 | -234.20±12.66 |
|  | 90% CI for LS mean | (-269.98, -189.66) | (-255.09, -213.31) |
|  | *p-*value^a^ | <0.0001 | <0.0001 |
|  | *p-*value^b^ | 0.8735 | |
| Month 2 | Mean±SD | 86.84±48.78 | 86.43±37.00 |
| Change from baseline | LS mean±SE | -231.43±24.33 | -245.11±12.66 |
|  | 90% CI for LS mean | (-271.59, -191.27) | (-266.00, -224.22) |
|  | *p-*value^a^ | <0.0001 | <0.0001 |
|  | *p-*value^b^ | 0.6192 | |
| Month 4 | Mean±SD | 75.45±45.57 | 111.88±60.03 |
| Change from baseline | LS mean±SE | -242.82±24.33 | -219.67±12.66 |
|  | 90% CI for LS mean | (-282.98, -202.66) | (-240.56, -198.78) |
|  | *p-*value^a^ | <0.0001 | <0.0001 |
|  | *p-*value^b^ | 0.4005 | |
| Month 6 | Mean±SD | 63.58±30.31 | 93.82±52.15 |
| Change from baseline | LS mean±SE | -254.69±24.33 | -240.05±12.95 |
|  | 90% CI for LS mean | (-294.85, -214.53) | (-261.42, -218.68) |
|  | *p-*value^a^ | <0.0001 | <0.0001 |
|  | *p-*value^b^ | 0.5968 | |
| Month 9 | Mean±SD | 87.63±60.62 | 91.21±40.25 |
| Change from baseline | LS mean±SE | -230.65±24.33 | -240.34±12.66 |
|  | 90% CI for LS mean | (-270.81, -190.49) | (-261.23, -219.45) |
|  | *p-*value^a^ | <0.0001 | <0.0001 |
|  | *p-*value^b^ | 0.7247 | |
| Month 12 | Mean±SD | 94.78±38.40 | 95.19±88.43 |
| Change from baseline | LS mean±SE | -223.49±24.33 | -236.36±12.66 |
|  | 90% CI for LS mean | (-263.65, -183.33) | (-257.25, -215.47) |
|  | *p-*value^a^ | <0.0001 | <0.0001 |
|  | *p-*value^b^ | 0.6402 | |
| Month 16 | Mean±SD | 70.90±32.31 | 96.57±71.88 |
| Change from baseline | LS mean±SE | -247.37±24.33 | -234.98±12.66 |
|  | 90% CI for LS mean | (-287.53, -207.21) | (-255.87, -214.09) |
|  | *p-*value^a^ | <0.0001 | <0.0001 |
|  | *p-*value^b^ | 0.6526 | |
| Month 20 | Mean±SD | 68.72±42.58 | 91.93±56.02 |
| Change from baseline | LS mean±SE | -249.56±24.33 | -243.36±13.26 |
|  | 90% CI for LS mean | (-289.72, -209.40) | (-265.25, -221.47) |
|  | *p-*value^a^ | <0.0001 | <0.0001 |
|  | *p-*value^b^ | 0.8238 | |
| Month 24 | Mean±SD | 94.00±41.75 | 86.09±63.43 |
| Change from baseline | LS mean±SE | -210.18±26.66 | -247.50±13.97 |
|  | 90% CI for LS mean | (-254.19, -166.18) | (-270.55, -224.45) |
|  | *p-*value^a^ | <0.0001 | <0.0001 |
|  | *p-*value^b^ | 0.2174 | |

^a^*p-*value calculated from the mixed-model for repeated measure testing within-group change from baseline. ^b^*p-*value calculated from the mixed-model for repeated measure testing between-group change from baseline. ^c^Baseline measurements at the extension study were treated as values at Month 12.

6-MWT, 6-minute walk test; CI, confidence interval; LS, least-square; SD, standard deviation; SE, standard error of mean.

**Table S9** Percent change from baseline in urine total GAG

| **Unit: %** | | **Switch**  **(n=6)** | **Maintenance**  **(n=22)** |
| --- | --- | --- | --- |
| Week 2 |  |  |  |
| Change from baseline | LS mean±SE | -65.40±7.70 | -63.69±4.10 |
|  | 90% CI for LS mean | (-78.11, -52.68) | (-70.46, -56.92) |
|  | *p-*value^a^ | <0.0001 | <0.0001 |
|  | *p-*value^b^ | 0.8451 | |
| Month 1 |  |  |  |
| Change from baseline | LS mean±SE | -68.96±7.70 | -70.27±4.01 |
|  | 90% CI for LS mean | (-81.67, -56.24) | (-76.89, -63.65) |
|  | *p-*value^a^ | <0.0001 | <0.0001 |
|  | *p-*value^b^ | 0.8801 | |
| Month 2 |  |  |  |
| Change from baseline | LS mean±SE | -69.88±7.70 | -73.51±4.01 |
|  | 90% CI for LS mean | (-82.60, -57.17) | (-80.13, -66.89) |
|  | *p-*value^a^ | <0.0001 | <0.0001 |
|  | *p-*value^b^ | 0.6772 | |
| Month 4 |  |  |  |
| Change from baseline | LS mean±SE | -74.70±7.70 | -66.36±4.01 |
|  | 90% CI for LS mean | (-87.41, -61.98) | (-72.98, -59.74) |
|  | *p-*value^a^ | <0.0001 | <0.0001 |
|  | *p-*value^b^ | 0.3392 | |
| Month 6 |  |  |  |
| Change from baseline | LS mean±SE | -77.92±7.70 | -70.64±4.10 |
|  | 90% CI for LS mean | (-90.64, -65.21) | (-77.41, -63.87) |
|  | *p-*value^a^ | <0.0001 | <0.0001 |
|  | *p-*value^b^ | 0.4059 | |
| Month 9 |  |  |  |
| Change from baseline | LS mean±SE | -70.51±7.70 | -72.13±4.01 |
|  | 90% CI for LS mean | (-83.22, -57.79) | (-78.75, -65.51) |
|  | *p-*value^a^ | <0.0001 | <0.0001 |
|  | *p-*value^b^ | 0.8523 | |
| Month 12 |  |  |  |
| Change from baseline | LS mean±SE | -65.49±7.70 | -68.75±4.01 |
|  | 90% CI for LS mean | (-78.21, -52.78) | (-75.37, -62.13) |
|  | *p-*value^a^ | <0.0001 | <0.0001 |
|  | *p-*value^b^ | 0.7081 | |
| Month 16 |  |  |  |
| Change from baseline | LS mean±SE | -72.86±7.70 | -69.88±4.01 |
|  | 90% CI for LS mean | (-85.57, -60.14) | (-76.50, -63.26) |
|  | *p-*value^a^ | <0.0001 | <0.0001 |
|  | *p-*value^b^ | 0.7329 | |
| Month 20 |  |  |  |
| Change from baseline | LS mean±SE | -74.83±7.70 | -73.46±4.20 |
|  | 90% CI for LS mean | (-87.54, -62.11) | (-80.39, -66.53) |
|  | *p-*value^a^ | <0.0001 | <0.0001 |
|  | *p-*value^b^ | 0.8769 | |
| Month 24 |  |  |  |
| Change from baseline | LS mean±SE | -59.38±8.43 | -73.14±4.42 |
|  | 90% CI for LS mean | (-73.29, -45.46) | (-80.43, -65.84) |
|  | *p-*value^a^ | <0.0001 | <0.0001 |
|  | *p-*value^b^ | 0.1502 | |

^a^*p-*value calculated from the mixed-model for repeated measure testing within-group change from baseline. ^b^*p-*value calculated from the mixed-model for repeated measure testing between-group change from baseline. ^c^Baseline measurements at the extension study were treated as values at Month 12.

6-MWT, 6-minute walk test; CI, confidence interval; LS, least-square; SD, standard deviation; SE, standard error of mean.

**Table S10** Observed mean at each visit and absolute change from baseline in urine dermatan sulfate

| **Unit: mg GAG/g Creatinine** | | **Switch**  **(n=6)** | **Maintenance**  **(n=22)** |
| --- | --- | --- | --- |
| Baseline | Mean±SD | 223.43±106.61 | 219.74±62.55 |
| Week 2 | Mean±SD | 85.05±34.56 | 66.56±20.24 |
| Change from baseline | LS mean±SE | -137.05±11.58 | -154.15±6.13 |
|  | 90% CI for LS mean | (-156.17, -117.93) | (-164.27, -144.03) |
|  | *p-*value^a^ | <0.0001 | <0.0001 |
|  | *p-*value^b^ | 0.1951 | |
| Month 1 | Mean±SD | 51.57±23.94 | 43.51±15.55 |
| Change from baseline | LS mean±SE | -170.53±11.58 | -176.98±6.01 |
|  | 90% CI for LS mean | (-189.65, -151.41) | (-186.91, -167.06) |
|  | *p-*value^a^ | <0.0001 | <0.0001 |
|  | *p-*value^b^ | 0.6230 | |
| Month 2 | Mean±SD | 56.32±35.66 | 36.49±16.07 |
| Change from baseline | LS mean±SE | -165.78±11.58 | -184.01±6.01 |
|  | 90% CI for LS mean | (-184.90, -146.66) | (-193.94, -174.09) |
|  | *p-*value^a^ | <0.0001 | <0.0001 |
|  | *p-*value^b^ | 0.1655 | |
| Month 4 | Mean±SD | 55.68±37.60 | 42.59±21.86 |
| Change from baseline | LS mean±SE | -166.41±11.58 | -177.90±6.01 |
|  | 90% CI for LS mean | (-185.53, -147.30) | (-187.83, -167.98) |
|  | *p-*value^a^ | <0.0001 | <0.0001 |
|  | *p-*value^b^ | 0.3817 | |
| Month 6 | Mean±SD | 39.82±20.46 | 31.70±18.46 |
| Change from baseline | LS mean±SE | -182.28±11.58 | -189.83±6.13 |
|  | 90% CI for LS mean | (-201.40, -163.16) | (-199.95, -179.71) |
|  | *p-*value^a^ | <0.0001 | <0.0001 |
|  | *p-*value^b^ | 0.5668 | |
| Month 9 | Mean±SD | 55.68±40.29 | 28.76±17.17 |
| Change from baseline | LS mean±SE | -166.41±11.58 | -191.74±6.01 |
|  | 90% CI for LS mean | (-185.53, -147.30) | (-201.66, -181.81) |
|  | *p-*value^a^ | <0.0001 | <0.0001 |
|  | *p-*value^b^ | 0.0545 | |
| Month 12 | Mean±SD | 49.16±32.49 | 29.71±29.39 |
| Change from baseline | LS mean±SE | -172.94±11.58 | -190.79±6.01 |
|  | 90% CI for LS mean | (-192.06, -153.82) | (-200.71, -180.86) |
|  | *p-*value^a^ | <0.0001 | <0.0001 |
|  | *p-*value^b^ | 0.1746 | |
| Month 16 | Mean±SD | 49.88±27.75 | 28.88±20.84 |
| Change from baseline | LS mean±SE | -172.21±11.58 | -191.62±6.01 |
|  | 90% CI for LS mean | (-191.33, -153.10) | (-201.55, -181.70) |
|  | *p-*value^a^ | <0.0001 | <0.0001 |
|  | *p-*value^b^ | 0.1400 | |
| Month 20 | Mean±SD | 34.04±27.52 | 25.29±21.71 |
| Change from baseline | LS mean±SE | -188.06±11.58 | -194.41±6.26 |
|  | 90% CI for LS mean | (-207.18, -168.94) | (-204.74, -184.08) |
|  | *p-*value^a^ | <0.0001 | <0.0001 |
|  | *p-*value^b^ | 0.6314 | |
| Month 24 | Mean±SD | 32.66±27.87 | 19.89±16.16 |
| Change from baseline | LS mean±SE | -191.66±12.53 | -200.62±6.55 |
|  | 90% CI for LS mean | (-212.34, -170.97) | (-211.43, -189.82) |
|  | *p-*value^a^ | <0.0001 | <0.0001 |
|  | *p-*value^b^ | 0.5280 | |

^a^*p-*value calculated from the mixed-model for repeated measure testing within-group change from baseline. ^b^P-value calculated from the mixed-model for repeated measure testing between-group change from baseline. ^c^Baseline measurements at the extension study were treated as values at Month 12.

6-MWT, 6-minute walk test; CI, confidence interval; LS, least-square; SD, standard deviation; SE, standard error of mean.

**Table S11** Percent change from baseline in urine dermatan sulfate

| **Unit: %** | | **Switch**  **(n=6)** | **Maintenance**  **(n=22)** |
| --- | --- | --- | --- |
| Week 2 |  |  |  |
| Change from baseline | LS mean±SE | -60.67±3.98 | -68.45±2.10 |
|  | 90% CI for LS mean | (-67.24, -54.10) | (-71.91, -64.99) |
|  | *p-*value^a^ | <0.0001 | <0.0001 |
|  | *p-*value^b^ | 0.0866 | |
| Month 1 |  |  |  |
| Change from baseline | LS mean±SE | -77.11±3.98 | -79.25±2.06 |
|  | 90% CI for LS mean | (-83.68, -70.54) | (-82.66, -75.85) |
|  | *p-*value^a^ | <0.0001 | <0.0001 |
|  | *p-*value^b^ | 0.6356 | |
| Month 2 |  |  |  |
| Change from baseline | LS mean±SE | -74.82±3.98 | -82.55±2.06 |
|  | 90% CI for LS mean | (-81.39, -68.25) | (-85.95, -79.15) |
|  | *p-*value^a^ | <0.0001 | <0.0001 |
|  | *p-*value^b^ | 0.0875 | |
| Month 4 |  |  |  |
| Change from baseline | LS mean±SE | -75.20±3.98 | -80.15±2.06 |
|  | 90% CI for LS mean | (-81.77, -68.63) | (-83.56, -76.75) |
|  | *p-*value^a^ | <0.0001 | <0.0001 |
|  | *p-*value^b^ | 0.2728 | |
| Month 6 |  |  |  |
| Change from baseline | LS mean±SE | -81.51±3.98 | -84.14±2.10 |
|  | 90% CI for LS mean | (-88.07, -74.94) | (-87.60, -80.68) |
|  | *p-*value^a^ | <0.0001 | <0.0001 |
|  | *p-*value^b^ | 0.5607 | |
| Month 9 |  |  |  |
| Change from baseline | LS mean±SE | -75.16±3.98 | -86.59±2.06 |
|  | 90% CI for LS mean | (-81.73, -68.59) | (-90.00, -83.19) |
|  | *p-*value^a^ | <0.0001 | <0.0001 |
|  | *p-*value^b^ | 0.0118 | |
| Month 12 |  |  |  |
| Change from baseline | LS mean±SE | -78.15±3.98 | -86.59±2.06 |
|  | 90% CI for LS mean | (-84.72, -71.59) | (-89.99, -83.18) |
|  | *p-*value^a^ | <0.0001 | <0.0001 |
|  | *p-*value^b^ | 0.0624 | |
| Month 16 |  |  |  |
| Change from baseline | LS mean±SE | -75.99±3.98 | -86.46±2.06 |
|  | 90% CI for LS mean | (-82.55, -69.42) | (-89.86, -83.05) |
|  | *p-*value^a^ | <0.0001 | <0.0001 |
|  | *p-*value^b^ | 0.0209 | |
| Month 20 |  |  |  |
| Change from baseline | LS mean±SE | -85.70±3.98 | -87.82±2.14 |
|  | 90% CI for LS mean | (-92.27, -79.13) | (-91.35, -84.29) |
|  | *p-*value^a^ | <0.0001 | <0.0001 |
|  | *p-*value^b^ | 0.6408 | |
| Month 24 |  |  |  |
| Change from baseline | LS mean±SE | -86.78±4.27 | -89.58±2.22 |
|  | 90% CI for LS mean | (-93.82, -79.74) | (-93.25, -85.91) |
|  | *p-*value^a^ | <0.0001 | <0.0001 |
|  | *p-*value^b^ | 0.5628 | |

^a^*p-*value calculated from the mixed-model for repeated measure testing within-group change from baseline. ^b^*p-*value calculated from the mixed-model for repeated measure testing between-group change from baseline. ^c^Baseline measurements at the extension study were treated as values at Month 12.

6-MWT, 6-minute walk test; CI, confidence interval; LS, least-square; SD, standard deviation; SE, standard error of mean.

**Table S12** Observed mean at each visit and absolute change from baseline in urine heparan sulfate

| **Unit: mg GAG/g Creatinine** | | **Switch**  **(n=6)** | **Maintenance**  **(n=22)** |
| --- | --- | --- | --- |
| Baseline | Mean±SD | 558.10±242.25 | 763.75±286.26 |
| Week 2 | Mean±SD | 182.27±47.59 | 241.73±94.61 |
| Change from baseline | LS mean±SE | -515.75±38.66 | -485.89±20.51 |
|  | 90% CI for LS mean | (-579.55, -451.95) | (-519.74, -452.03) |
|  | *p-*value^a^ | <0.0001 | <0.0001 |
|  | *p-*value^b^ | 0.4971 | |
| Month 1 | Mean±SD | 108.02±44.93 | 154.15±59.65 |
| Change from baseline | LS mean±SE | -590.00±38.66 | -573.90±20.07 |
|  | 90% CI for LS mean | (-653.80, -526.19) | (-607.02, -540.78) |
|  | *p-*value^a^ | <0.0001 | <0.0001 |
|  | *p-*value^b^ | 0.7130 | |
| Month 2 | Mean±SD | 123.65±64.50 | 130.51±50.43 |
| Change from baseline | LS mean±SE | -574.37±38.66 | -597.54±20.07 |
|  | 90% CI for LS mean | (-638.17, -510.56) | (-630.66, -564.41) |
|  | *p-*value^a^ | <0.0001 | <0.0001 |
|  | *p-*value^b^ | 0.5966 | |
| Month 4 | Mean±SD | 113.70±59.93 | 138.76±62.11 |
| Change from baseline | LS mean±SE | -584.32±38.66 | -589.29±20.07 |
|  | 90% CI for LS mean | (-648.12, -520.51) | (-622.42, -556.17) |
|  | *p-*value^a^ | <0.0001 | <0.0001 |
|  | *p-*value^b^ | 0.9095 | |
| Month 6 | Mean±SD | 94.92±41.46 | 109.01±61.59 |
| Change from baseline | LS mean±SE | -603.10±38.66 | -622.08±20.52 |
|  | 90% CI for LS mean | (-666.90, -539.30) | (-655.95, -588.22) |
|  | *p-*value^a^ | <0.0001 | <0.0001 |
|  | *p-*value^b^ | 0.6660 | |
| Month 9 | Mean±SD | 126.67±88.30 | 88.40±45.60 |
| Change from baseline | LS mean±SE | -571.35±38.66 | -639.65±20.07 |
|  | 90% CI for LS mean | (-635.15, -507.55) | (-672.77, -606.52) |
|  | *p-*value^a^ | <0.0001 | <0.0001 |
|  | *p-*value^b^ | 0.1194 | |
| Month 12 | Mean±SD | 109.31±59.36 | 92.93±86.07 |
| Change from baseline | LS mean±SE | -588.71±38.66 | -635.12±20.07 |
|  | 90% CI for LS mean | (-652.52, -524.91) | (-668.24, -602.00) |
|  | *p-*value^a^ | <0.0001 | <0.0001 |
|  | *p-*value^b^ | 0.2894 | |
| Month 16 | Mean±SD | 124.61±68.60 | 94.65±55.06 |
| Change from baseline | LS mean±SE | -573.40±38.66 | -633.40±20.07 |
|  | 90% CI for LS mean | (-637.21, -509.60) | (-666.52, -600.28) |
|  | *p-*value^a^ | <0.0001 | <0.0001 |
|  | *p-*value^b^ | 0.1711 | |
| Month 20 | Mean±SD | 104.15±78.71 | 82.23±55.20 |
| Change from baseline | LS mean±SE | -593.87±38.66 | -646.93±21.02 |
|  | 90% CI for LS mean | (-657.68, -530.07) | (-681.61, -612.25) |
|  | *p-*value^a^ | <0.0001 | <0.0001 |
|  | *p-*value^b^ | 0.2308 | |
| Month 24 | Mean±SD | 87.73±63.38 | 72.80±56.53 |
| Change from baseline | LS mean±SE | -613.50±42.28 | -657.08±22.12 |
|  | 90% CI for LS mean | (-683.28, -543.73) | (-693.58, -620.57) |
|  | *p-*value^a^ | <0.0001 | <0.0001 |
|  | *p-*value^b^ | 0.3640 | |

^a^*p-*value calculated from the mixed-model for repeated measure testing within-group change from baseline. ^b^*p-*value calculated from the mixed-model for repeated measure testing between-group change from baseline. ^c^Baseline measurements at the extension study were treated as values at Month 12.

6-MWT, 6-minute walk test; CI, confidence interval; LS, least-square; SD, standard deviation; SE, standard error of mean.

**Table S13** Percent change from baseline in urine heparan sulfate

| **Unit: %** | | **Switch**  **(n=6)** | **Maintenance**  **(n=22)** |
| --- | --- | --- | --- |
| Week 2 |  |  |  |
| Change from baseline | LS mean±SE | -65.86±3.55 | -66.76±1.87 |
|  | 90% CI for LS mean | (-71.71, -60.00) | (-69.85, -63.66) |
|  | *p-*value^a^ | <0.0001 | <0.0001 |
|  | *p-*value^b^ | 0.8235 | |
| Month 1 |  |  |  |
| Change from baseline | LS mean±SE | -80.95±3.55 | -78.73±1.84 |
|  | 90% CI for LS mean | (-86.81, -75.09) | (-81.77, -75.70) |
|  | *p-*value^a^ | <0.0001 | <0.0001 |
|  | *p-*value^b^ | 0.5821 | |
| Month 2 |  |  |  |
| Change from baseline | LS mean±SE | -78.36±3.55 | -81.70±1.84 |
|  | 90% CI for LS mean | (-84.21, -72.50) | (-84.74, -78.67) |
|  | *p-*value^a^ | <0.0001 | <0.0001 |
|  | *p-*value^b^ | 0.4057 | |
| Month 4 |  |  |  |
| Change from baseline | LS mean±SE | -80.39±3.55 | -80.69±1.84 |
|  | 90% CI for LS mean | (-86.24, -74.53) | (-83.73, -77.65) |
|  | *p-*value^a^ | <0.0001 | <0.0001 |
|  | *p-*value^b^ | 0.9404 | |
| Month 6 |  |  |  |
| Change from baseline | LS mean±SE | -83.57±3.55 | -84.79±1.87 |
|  | 90% CI for LS mean | (-89.42, -77.71) | (-87.89, -81.70) |
|  | *p-*value^a^ | <0.0001 | <0.0001 |
|  | *p-*value^b^ | 0.7616 | |
| Month 9 |  |  |  |
| Change from baseline | LS mean±SE | -79.36±3.55 | -87.50±1.84 |
|  | 90% CI for LS mean | (-85.22, -73.50) | (-90.54, -84.46) |
|  | *p-*value^a^ | <0.0001 | <0.0001 |
|  | *p-*value^b^ | 0.0438 | |
| Month 12 |  |  |  |
| Change from baseline | LS mean±SE | -81.36±3.55 | -86.67±1.84 |
|  | 90% CI for LS mean | (-87.22, -75.50) | (-89.71, -83.64) |
|  | *p-*value^a^ | <0.0001 | <0.0001 |
|  | *p-*value^b^ | 0.1871 | |
| Month 16 |  |  |  |
| Change from baseline | LS mean±SE | -78.72±3.55 | -86.28±1.84 |
|  | 90% CI for LS mean | (-84.57, -72.86) | (-89.32, -83.24) |
|  | *p-*value^a^ | <0.0001 | <0.0001 |
|  | *p-*value^b^ | 0.0609 | |
| Month 20 |  |  |  |
| Change from baseline | LS mean±SE | -83.70±3.55 | -87.78±1.91 |
|  | 90% CI for LS mean | (-89.56, -77.84) | (-90.93, -84.63) |
|  | *p-*value^a^ | <0.0001 | <0.0001 |
|  | *p-*value^b^ | 0.3148 | |
| Month 24 |  |  |  |
| Change from baseline | LS mean±SE | -86.30±3.82 | -88.01±1.99 |
|  | 90% CI for LS mean | (-92.60, -80.01) | (-91.30, -84.73) |
|  | *p-*value^a^ | <0.0001 | <0.0001 |
|  | *p-*value^b^ | 0.6926 | |

^a^*p-*value calculated from the mixed-model for repeated measure testing within-group change from baseline. ^b^*p-*value calculated from the mixed-model for repeated measure testing between-group change from baseline. ^c^Baseline measurements at the extension study were treated as values at Month 12.

6-MWT, 6-minute walk test; CI, confidence interval; LS, least-square; SD, standard deviation; SE, standard error of mean.

**Table S14** Incidence of adverse events by neutralizing antibody formation status and genotype type

| **Neutralizing antibodies** | **Positive**  **(n=16)** | **Negative**  **(n=12)** |
| --- | --- | --- |
| **Genotype group 1^a^** | **n=5** | **n=4** |
| Any adverse events | 5 (100.0) [55] | 4 (100.0) [26] |
| Serious adverse events | 2 (40.0) [4] | 0 (0.0) [0] |
| Acute adverse events | 1 (20.0) [1] | 0 (0.0) [0] |
| **Genotype group 2^b^** | **n=11** | **n=8** |
| Any adverse events | 11 (100.0) [166] | 8 (100.0) [76] |
| Serious adverse events | 5 (45.5) [5] | 1 (12.5) [2] |
| Acute adverse events | 6 (54.5) [46] | 1 (12.5) [12] |

Data are number of patients (%) with the number of events in the square brackets. ^a^Missense type. ^b^All other variants, excluding missense variants.

**Table S15** Percent changes at Month 24 from baseline in efficacy outcomes by neutralizing antibody development status

| **Neutralizing antibodies** | **Positive (n=16)** | **Negative (n=12)** | **Difference (90% CI)** |
| --- | --- | --- | --- |
| **6-MWT** |  |  |  |
| Mean±SD | 29.12±14.23 | 16.34±8.82 | 12.77±12.24 (4.80, 20.74) |
| *p-*value | <0.0001^a^ | 0.0010^a^ | 0.0087^b^ |
| **uGAG** |  |  |  |
| Mean±SD | -67.21±24.78 | 75.03±15.06 | 7.83±21.22 (-6.00, 21.65) |
| *p-*value | <0.0001^a^ | 0.0005^a^ | 0.5934^b^ |

^a^*p-*value for within-group change from baseline; ^b^*p-*value for between-group (neutralizing antibody positive vs. negative) comparison for the change from baseline.

6-MWT, 6-minute walk test; CI, confidence interval; standard deviation; uGAG, urine glycosaminoglycans.

**Table S16** Percent changes at Month 24 from baseline in efficacy outcomes by genotype

| **Genotype group** | **Group 1^a^ (N=9)** | **Group 2^b^ (N=19)** | **Difference (90% CI)** |
| --- | --- | --- | --- |
| **6-MWT** |  |  |  |
| Mean±SD | 25.13±14.87 | 22.94±13.36 | 2.20±13.84 (-7.36, 11.75) |
| *p-*value | 0.0039^c^ | <0.0001^c^ | 0.8827^d^ |
| **uGAG** |  |  |  |
| Mean±SD | -74.65±10.39 | -68.63±24.77 | -6.02±21.40 (-17.37, 5.33) |
| p-value | 0.0039^c^ | <0.0001^c^ | 0.9216^d^ |

^a^Missense type. ^b^All other variants excluding missense variants. ^c^*p-*value for within-group change from baseline; ^d^*p-*value for between-group (genotype group 1 vs. group 2) comparison for the change from baseline.

6-MWT, 6-minute walk test; CI, confidence interval; SD, standard deviation; uGAG, urine glycosaminoglycans.

**Fig. S1** Visualization of individual patient’s percent change in 6-minute walk test (6-MWT) at Month 24 from baseline. The labels on the X-axis in graphs A and B indicate individual age (y, years). If multiple participants had the same age, a consecutive number was added to the label, for example, 6y1, 6y2, and 6y3.

**Fig. S2** Visualization of individual patient’s immunogenicity results. Week 0 was the baseline. Participants in the switch group are marked with a star after the anonymized identifier, and the rest are those enrolled in the maintenance group. A red minus symbol denotes a negative result (antibody level undetectable) from each test whereas the blue plus symbol denotes a positive result (antibody detected).
